# An Injectable Hybrid Gelatin Methacryloyl (GelMA)/Phenyl Isothiocyanate-Modified Gelatin (Gel-Phe) Bioadhesive for Oral/Dental Hemostasis Applications

**DOI:** 10.3390/polym13142386

**Published:** 2021-07-20

**Authors:** Wan-Chun Chang, Au-Zou Tai, Nian-Yun Tsai, Yi-Chen Ethan Li

**Affiliations:** 1 Department of Chemical Engineering, Feng Chia University, Taichung 40724, Taiwan; d0642564@mail.fcu.edu.tw (W.-C.C.); lilly95024@gmail.com (N.-Y.T.); 2 Ph.D. Program of Mechanical and Aeronautical Engineering, Feng Chia University, Taichung 40724, Taiwan; bensonoj1021@gmail.com

**Keywords:** phenyl isothiocyanate, gelatin, GelMA: photo-crosslink, injectable hydrogel, hemostasis

## Abstract

Biomaterials are widely used for effectively controlling bleeding in oral/dental surgical procedures. Here, gelatin methacryloyl (GelMA) was synthesized by grafting methacrylic anhydride on gelatin backbone, and phenyl isothiocyanate-modified gelatin (Gel-Phe) was synthesized by conjugating different gelatin/phenyl isothiocyanate molar ratios (G/P ratios) (i.e., 1:1, 1:5, 1:10, 1:15, 1:25, 1:50, 1:100, and 1:150) with gelatin polymer chains. Afterward, we combined GelMA and Gel-Phe as an injectable and photo-crosslinkable bioadhesive. This hybrid material system combines photo-crosslinking chemistry and supramolecular interactions for the design of bioadhesives exhibiting a highly porous structure, injectability, and regulable mechanical properties. By simply regulating the G/P ratio (1:1–1:15) and UV exposure times (15–60 s), it was possible to modulate the injectability and mechanical properties of the GelMA/Gel-Phe bioadhesive. Moreover, we demonstrated that the GelMA/Gel-Phe bioadhesive showed low cytotoxicity, a highly porous network, and the phenyl-isothiourea and amine residues on Gel-Phe and GelMA polymers with synergized hemostatic properties towards fast blood absorption and rapid clotting effect. An in vitro porcine skin bleeding and an in vitro dental bleeding model confirmed that the bioadhesive could be directly extruded into the bleeding site, rapidly photo-crosslinked, and reduced blood clotting time by 45%. Moreover, the in situ crosslinked bioadhesive could be easily removed from the bleeding site after clotting, avoiding secondary wound injury. Overall, this injectable GelMA/Gel-Phe bioadhesive stands as a promising hemostatic material in oral/dental surgical procedures.

## 1. Introduction

Excessive hemorrhage complicates surgical processes and increases the mortality rate. Hemostatic control plays a critical role in all surgical procedures [1]. In an oral/dental surgical procedure, continuous blood oozing is normal but requires attention that may sometimes interrupt the procedure [2]. Additionally, Shaikh et al. reported that controlling continuous blood oozing, the space for blood clots, and blood clot stabilization are highly relative to dental wound healing after surgical procedure [3]. They also mentioned that a thick blood clot might induce poor plasmatic circulation and affect the healing between graft and gingival tissues at the initial healing stage [3]. The information indicated the critical role of controlling bleeding or blood clot formation. In clinical oral/dental surgery, patients tightly biting on a cotton ball placed on the extraction site is a gold-standard method to control bleeding after dental surgery. Sometimes, local hemostasis during tooth extraction or oral surgical procedures may be difficult in some particular cases. For instance, patients are advised to stop taking anticoagulants for several days before tooth extraction or surgery if they regularly take such a prescription drug. However, patients suspending use of anticoagulants may suffer from severe cardiovascular issues such as thromboembolism [4]. Although the traditional techniques show the proper hemostatic effects for dental surgical procedures, a broad range of hemostatic materials have been developed to assist the efficiency of hemostasis. For instance, BioGlue and bone wax are widely used to seal wounds and perform the hemostatic process [5]. One of the BioGlue components, glutaraldehyde, could crosslink the cells around wounds, kill cells, and compromise the regrowth of new tissues [6]. Similarly, bone wax is a paraffin-based material used for bone bleeding during tooth extraction, but the non-resorbable property of bone wax may affect osteogenesis [7]. Therefore, these non-resorbable sealants may produce undesired side effects on soft tissues in oral surgery.

Recently, adsorbable and biocompatible materials have been widely used to control blood bleeding. Ito et al. developed a hybrid poly(acrylic acid) and poly(vinylpyrrolidone) sponge for tooth extraction or oral surgical procedures. After contacting blood, the hybrid sponge with a high water-swellable property swells and enables it to stick on the hemorrhaging spot and stop bleeding [8]. In addition to synthetic materials, using natural biomimetic materials for hemostasis is another strategy in dental surgical processes. For instance, collagen- and cellulose-based sponges provide a highly absorbable and water-containing property, which can soak up a large volume of liquid, induce the aggregation of platelets for blood clotting, and rapidly degrade within several weeks [9,10]. Despite the porous and three-dimensional structure of sponges promoting blood clotting, soft and injectable materials such as hemostatic hydrogels have attracted research interest within the past decade. Previous studies have reported that the hemostatic hydrogels possess the unique ability to adequately fill and adhere to irregular wounds, contributing to more efficient hemorrhage control [11,12,13]. Moreover, soft hemostatic hydrogels could be easily removed through water or phosphate buffered saline (PBS), which can be carried out to reduce secondary wound bleeding [14]. As such, soft hemostatic hydrogels show the potential to be a candidate for hemostatic applications in tooth extraction or dental surgical procedures.

Many studies have shown various materials such as fibrin-/silk- [15], polysaccharide- [16], and collagen-based adhesives [17], which can be used as soft hydrogels for hemostasis. Collagen is one of the essential extracellular matrices in gingival tissues [18]. As such, collagen-based soft hydrogels have been an option for use in oral/dental hemostasis. However, several previous studies have reported that collagen and related collagen-based products from animal sources may induce allergic reactions [19,20].

Gelatin is a natural polymer obtained from denatured collagen. Gelatin’s structure, similar to collagen, endows it with biocompatible and biodegradable properties—such as bioadhesives, drug carriers, or substrates in biomedical applications [21,22,23]. Moreover, a gelatin hydrogel offers a hemostatic function because it could swell through the absorption of blood, promote the aggregation of platelets, and then activate the platelets to accelerate the blood clotting [24]. Despite these apparent hemostatic effects from gelatin, a gelatin hydrogel presents poor mechanical properties and low gelation ability under physiological temperature and wet conditions [25]. A methacrylation method provides gelatin with a strategy to improve the mechanical properties of gelatin. A photo-crosslinkable polymer, gelatin methacryloyl (GelMA), has also been widely applied in biomedical engineering [26,27,28]. The synthesized GelMA has been confirmed for its good biocompatibility and low immunogenicity [28]. Moreover, regulating the methacrylation degree on the gelatin backbone endows GelMA with a broad range of tunable stiffness (from 1 kPa to more than a hundred kPa) [26,29]. In addition to methacrylation, Feng et al. modified gelatin by grafting the small hydrophobic molecules on gelatin polymers [30]. The modified gelatin polymer shows the thermo-reversible, strong stretchable, and tough properties through the supramolecular and hydrophobic interactions among the polymer chains. Moreover, Fares et al. also developed a GelMA-based hybrid hydrogel with high mechanical strength [29], indicating that blending different components could produce superior synergistic effects for regulating the physicochemical properties of gelatin polymers.

In this study, we assume that combining the bioactivity of GelMA and the stretchable toughness of phenyl isothiocyanate-modified gelatin (Gel-Phe) would create a hybrid injectable bioadhesive with controllable injectability, porosity, and mechanical properties. Then, these factors may regulate the performance of hemostatic materials. The complementary chemical crosslink and supramolecular interactions of GelMA and Gel-Phe contribute a strategy to regulate the adhesivity, gel network, and hemostatic properties. Furthermore, we also envisioned that the supramolecular interactions on Gel-Phe and amine group on GelMA might offer synergistic effects on hemostatic properties. To validate our hypothesis, GelMA and Gel-Phe polymers were synthesized by modifying the polymer backbones with methacrylic anhydride (MA) and phenyl isothiocyanate (Phe). Then, the chemical functional groups were identified via Fourier-transform infrared spectroscopy (FTIR) and ^1^H-nuclear magnetic resonance (^1^H-NMR). The injectability of the GelMA/Gel-Phe bioadhesive was evaluated by using a rheometer and an extrusion 3D bioprinter. Subsequently, the porous structure of hybrid bioadhesives was evaluated using scanning electron microscopy (SEM). We also tested the mechanical properties of bioadhesives through compression testing and burst-pressure testing. Furthermore, the cell viability of hybrid bioadhesives was measured through an in vitro cell viability assay. Lastly, we evaluated the injectability of GelMA/Gel-Phe bioadhesive to support the hemostasis of the wound through two in vitro bleeding models.

## 2. Materials and Method

### 2.1. Materials

3-(4,5-dimethylthiazol-2-yl)-2,5-diphenyltetrazolium bromide (MTT), Phe, MA, gelatin from porcine skin, 2-Hydroxy-4′-(2-hydroxyethoxy)-2-methylpropiophenone (Irgacure 2959), dimethyl sulfoxide (DMSO), and calcium chloride (CaCl_2_) were purchased from Sigma-Aldrich (UNI-ONWARD, Corp., Taiwan). Fetal bovine serum (FBS), Dulbecco’s modified Eagle medium (DMEM), Mouse fibroblast cell line L929, and trypsin were purchased from the Thermo Fisher Scientific Inc. (Level Biotechnology Inc., Taichung, Taiwan).

### 2.2. Synthesis of GelMA and Gel-Phe Polymers

The protocol of GelMA synthesis was modified based on our previous work [31]. Briefly, MA was added into a 10% *w*/*v* gelatin solution. After 2 h of reaction, the solution was purified through a dialysis procedure for five days at 40 °C. Afterward, the GelMA solution was stored at −80 °C overnight and then freeze-dried for five days. The synthesis of Gel-Phe was slightly modified based on the previous study [30]. First, 10% (*w*/*v*) gelatin was dissolved in DMSO at 60 °C. Phe with different gelatin/phenyl isothiocyanate molar ratios (G/P ratios) (i.e., 1:1, 1:5, 1:10, 1:15, 1:25, 1:50, 1:100, and 1:150) was added to the 10% gelatin solution to react for five hours at 50 °C. The Gel-Phe solution was purified through the dialysis procedure in DI water for three days at room temperature (6 kDa cutoff dialysis membrane). The purified Gel-Phe was stored in DI water at −20 °C. Afterward, the purified Gel-Phe was freeze-dried to remove water for the characterization. The characterization of Gel-Phe and GelMA was carried out via FTIR spectroscopy (PerkinElmer, Akron, OH, USA), and ^1^H NMR was recorded with a 600 MHz spectrometer (Agilent Technologies, Santa Clara, CA, USA)

### 2.3. Evaluation of the Water Solubility, Gel Formation, and Rheological and Mechanical Properties of GelMA/Gel-Phe Bioadhesives

The water solubility of Gel-Phe was tested by immersing Gel-Phe into 1 mL water with or without GelMA. Then, the temperature of the Gel-Phe or GelMA/Gel-Phe solutions was increased to 50 °C and mixed vigorously. Afterward, the temperature of Gel-Phe or GelMA/Gel-Phe solutions was cooled to room temperature to observe the water solubility of Gel-Phe in the Gel-Phe or GelMA/Gel-Phe solutions.

The gel formation of GelMA/Gel-Phe bioadhesives was tested by performing the 15 s, 30 s, and 60 s UV exposure times for 5%GelMA/5%Gel-Phe and 10%GelMA/10%Gel-Phe solutions. The wavelength of UV light was at 365 nm for polymerization, and the intensity of UV light was 800 mW/cm^2^, which was controlled via a UV light source system (UV areacure, Brightek Co., Ltd., Taiwan). The GelMA/Gel-Phe solutions were loaded in a cylindric mold (outer diameter: 1.2 cm, inner diameter: 0.8 cm, and height: 0.3 cm) to form a cylinder-shaped hydrogel. After the exposure to UV light, the gels were carefully removing from the mold, and the crosslinked GelMA/Gel-Phe bioadhesives were used for the following tests.

The responses of 5%GelMA/5%Gel-Phe and 10%GelMA/10%Gel-Phe solutions with 0.5% Irgacure 2959 (i.e., photoinitiator (PI)) to shear to investigate the rheological properties were measured by a rheometer (DHR-20, TA Instrument, New Castle, DE, USA) through continuous flow experiments under a linearly ramped shear rate from 1 to 100 s^−1^. The hybrid bioadhesive was examined in injectability experiments, where oscillatory time sweeps were set with alternating high (250%) and low (0.5%) strains at 10 Hz every 2 min. Moreover, we also tested the injectability of the bioadhesives via a 3D printing technology. The window, grid, and circle shapes were designed by SolidWorks software (Dassault Systems, Waltham, MA, USA). Then, the bioadhesives were printed as the window, grid, and circle shapes by using a customized extrusion bioprinter modified from a Prusa i3 3D printer (Prusa, USA).

The crosslinked 10%GelMA/10%Gel-Phe/0.5%PI bioadhesives with the cylindric shape were immersed in PBS to remove the unpolymerized GelMA and PI and were further removed the excess water via a freeze-drying process. Then, the freeze-dried bioadhesives were stuck to the sample stage by using conductive copper tape. Afterward, a gold film was performed on the sample via vacuum spray, and then the structure of crosslinked GelMA/Gel-Phe bioadhesives was observed by using SEM (Hitachi, Japan).

The mechanical property of 10%GelMA/10%Gel-Phe/0.5%PI bioadhesives crosslinked by 15, 30, and 60 s of UV light exposure was evaluated at a rate of 20% strain per min on a texture analyzer (RapidTA, Horn Instruments Co., Ltd., Taichung, Taiwan). The compressive modulus was obtained from the slope of the linear region corresponding with 0–5% strain. The results of mechanical properties were collected from three independent experiments for each condition (*n* = 3/condition), and each condition had three samples for each independent experiment. Then, the results were analyzed via the following statistical analysis.

### 2.4. In Vitro Burst Model

The in vitro burst pressure of crosslinked 10%GelMA/10%Gel-Phe/0.5%PI bioadhesives was evaluated through a slightly modified protocol based on the previous works [32,33] and the standard ASTM F2392-04 method. The porcine skin sheet (4 cm × 4 cm, commercial from the food market) was treated with PBS before testing. A collagen sheet (Sigma-Aldrich, USA) with a circular defect with a 3 mm diameter in the center was sandwiched between two porcine skins sheets. Then, the defect was filled with hydrogels (thickness ≅ 1 mm). Afterward, the collagen sheet was removed and connected with the testing system. A continuous saline flow was infused through a pump, and we increased the pressure until the samples burst. The burst pressure was recorded via an absolute pressure/temperature sensor (PASCO, Roseville, CA, USA). The results of burst pressure were collected from three independent experiments for each condition (*n* = 3/condition), and each condition had three samples for each independent experiment. Then, the results were analyzed via the following statistical analysis.

### 2.5. Identification of the Cytotoxicity of GelMA/Gel-Phe Bioadhesives

L929 cells were cultured with DMEM containing 1% streptomycin, 1% penicillin, 10% FBS, 5% CO_2_, kept at 37 °C, and were subcultured every three days. The crosslinked GelMA/Gel-Phe bioadhesives (0.2 g/mL) were incubated in the culture medium to 37 °C for 24 h. Then, the extraction medium was used as a conditioned medium, and the cells were treated with the conditioned medium extracted from bioadhesives following the ISO10993 protocol. Then, cellular metabolic activity of cells was assessed by using MTT assay (Sigma-Aldrich). Cells were incubated with the MTT solution for 4 h at 37 °C. Afterward, the MTT reagent was removed, and the purple crystal was dissolved in DMSO. Then, the collected purple supernatant was measured the absorbance of the supernatant at 570 nm using a microplate reader (Epoch, BioTek, Winooski, VT, USA). The results of the MTT assay were collected from three independent experiments for each condition (*n* = 3/condition), and each condition had six samples for each independent experiment. Then, the results were analyzed via the following statistical analysis.

### 2.6. Blood Clotting Time Assay

According to the protocol of previous study [34], 1% solid content of GelMA/Gel-Phe bioadhesives per 1 mL blood was first placed in an Eppendorf tube. Then, the GelMA/Gel-Phe bioadhesives in the Eppendorf tube were treated with 1 ml commercial sheep blood (Taiwan Prepared Media, Taiwan) mixed with 0.02 M CaCl_2_ and followed by vortexing for 10 s. The blood coagulation behavior was evaluated at 37 °C, and the time was stopped when the blood beginning clotting. The blood clotting results were collected from three independent experiments for each condition (*n* = 3/condition), and each condition had three samples for each independent experiment. Then, the results were analyzed via the following statistical analysis.

### 2.7. In Vitro Porcine Skin and In Vitro Dental Bleeding Models

A protocol from our previous study provided an in vitro porcine skin bleeding model [35], which was designed to investigate the feasibility of the bioadhesives for blood coagulation. The tube was embedded in the porcine skin for perfusing a blood sample (400 μL/min). Then, the tube’s output was then covered with the hybrid GelMA/Gel-Phe bioadhesives on the bleeding site and treated with a UV light for crosslink simultaneously. Similar to the in vitro porcine skin bleeding model, an in vitro dental bleeding was designed by embedding a tube, which perfused a blood sample (400 μL/min) into the location of lower left third molar to mimic the bleeding after tooth extraction. Afterward, the tube’s output was then covered with the hybrid GelMA/Gel-Phe bioadhesives on the bleeding site and treated with a UV light for crosslink simultaneously.

### 2.8. Statistical Analysis

Statistical analysis in this study was performed using one-way ANOVA followed by Tukey’s test to compare data (* *p* < 0.05, ** *p* < 0.01).

## 3. Results and Discussion

### 3.1. Design of Hybrid GelMA/Gel-Phe Bioadhesives

In the past decade, bioadhesives have assumed a major role in developing hemostatic agents because of their operable property and rapid in situ gelation behavior, as well as the ability to attach onto irregular wounds, offering an effective alternative to traditional sponges and gauze-type hemostatic substrates in oral/dental surgical procedures [36].

**Figure 1:**
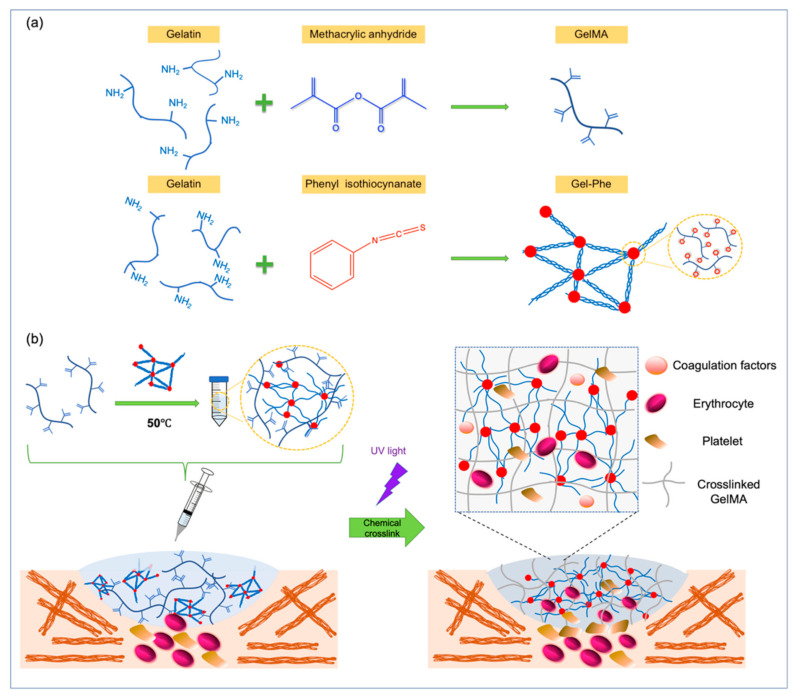
(**a**) Representation of the synthesis of GelMA and Gel-Ph, and (**b**) schematic illustration of an injectable GelMA/Gel-Phe bioadhesive crosslinked using UV light for hemostasis.

Many factors, including the physical properties (e.g., porosity), chemical functional groups (e.g., internal molecular interactions), and biological factors (e.g., proteins or peptides), can affect the hemostatic efficiency of bioadhesives [37,38,39,40]. This study developed a hybrid injectable bioadhesive with tunable mechanical properties and supramolecular interactions based on GelMA and Gel-Phe (Figure 1). The addition of a phenyl isothiocyanate group enhanced the supramolecular aromatic interactions between the molecular chains of Gel-Phe via π–interactions, enabling an increase in the viscosity of GelMA/Gel-Phe bioadhesive. Then, the bioadhesive were injected on the bleeding site and treated with UV light to quickly form a crosslinked hydrogel through the crosslinking chemistries from the methacrylated GelMA (Figure 1b). Afterward, the polymer chains of GelMA and Gel-Phe could interlace to form a network structure, contributing to adequate mechanical properties and porosity [41]. As such, the GelMA/Gel-Phe bioadhesive is characterized by bioactive sites and a high surface area via the mixture of the RGD peptide-binding motif from gelatin, hydrophobic interactions from Gel-Phe, and increased porous structures from the crisscross network structure from these two polymers [42,43]. Therefore, the blood could be rapidly absorbed and clotted when the GelMA/Gel-Phe bioadhesive was directly crosslinked on the bleeding site.

### 3.2. Synthesis and Characterization of GelMA/Gel-Phe

**Figure 2:**
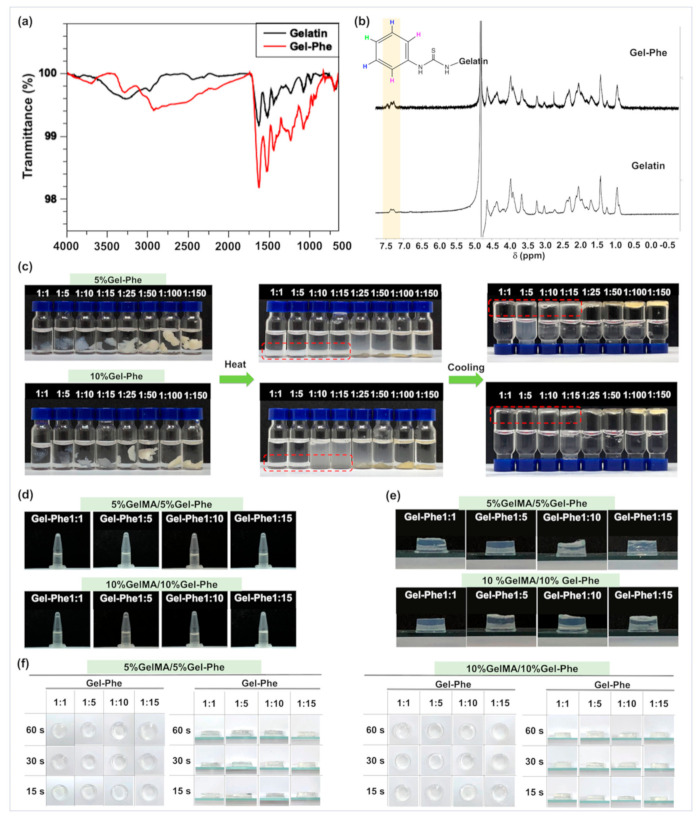
Characterization of gelatin and Gel-Phe. (**a**) The FTIR spectrum of gelatin and Gel-Phe showed characteristic relative peaks of functional groups. (**b**) The ^1^H-NMR spectrum of gelatin and Gel-Phe validated the chemical modification of phenyl isothiocyanate grafted on the gelatin backbones. (**c**) The morphologies of Gel-Phe with different G/P ratios immersed in water after the heating–cooling process. Optical imaging showed the gel formation of Gel-Phe with different G/P ratios mixed with GelMA and 0.5% PI (**d**) before and (**e**) after UV light exposure. (**f**) The overhead view and side-view images showed the crosslinked hydrogel morphologies of GelMA/Gel-Phe solutions with 0.5% PI by treatment with different UV light exposure times (15, 30, and 60 s).

To identify GelMA and Gel-Phe, we analyzed the functional groups on gelatin backbones with and without the chemical modifications through FTIR and ^1^H-NMR spectrometry. The FTIR analysis showed the reaction among the phenyl isothiocyanate and amine and hydroxyl groups on gelatin backbones (Figure 2a). The increased peaks of the FTIR spectrum at 3300, 3180, 1530, and 830 cm^−1^ related to the symmetric and asymmetric -NH, C–N, and C=S bonds were observed, respectively [44,45,46], indicating phenyl isothiocyanate was grafted on the gelatin backbones successfully. A previous study has reported that the methacrylation reaction of gelatin [47]. Similarly, the strongly increased peaks related to the amine, C=O, C=C, C–N, and C–O–C bonds were significantly observed at 3397, 1650, 1631, 1530, and 1063 cm^−1^ (Figure S1a). Next, the conjugation of phenyl isothiocyanate and methacrylate on gelatin backbones was identified via ^1^HNMR spectrometer (Figure 2b and Figure S1b). After the modification, the significant increase in the peaks at 7.1–7.3 ppm and 6.25, 5.75, and 1.90 ppm indicated the protons of the aromatic group on Gel-Phe and the protons of methacrylate groups and GelMA. Additionally, a significant decrease in peaks at 2.9 ppm related to the protons of methylene of lysine on raw gelatin was also observed. These changes of peaks in the ^1^HNMR spectrum were consistent with the results of Gel-Phe and GelMA from previous studies [30,47].

A previous study reported that the Gel-Phe adhesive possesses thermo-reversible and thermoplastic properties [30]. The Gel-Phe adhesive can transit into a highly viscous state when the temperature is higher than 45 °C. In contrast, the Gel-Phe adhesive is in a solid state with strong mechanical properties when the temperature is less than 45 °C. Moreover, this previous study also indicated that the Gel-Phe adhesive with poor water solubility, even the temperature higher than 45 °C [30]. Previous study showed that poor water solubility of materials may be unsuitable for use in biomedical applications. To enhance water solubility, we further symmetrically synthesized the Gel-Phe polymers with different G/P ratios (Figure S2) and evaluated the water solubility of these Gel-Phe polymers. As shown in Figure 2c, the Gel-Phe polymers were first immersed into water, the temperature was increased to 50 °C, and then subsequently cooled to room temperature. Interestingly, when the G/P ratio of Gel-Phe was decreased to less than 1:25, the G/P ratios of 1:15, 1:10, 1:5, and 1:1 enabled Gel-Phe to dissolve in water (red rectangle, Figure 2c). Afterward, the Gel-Phe polymers with the ratios of 1:15, 1:10, 1:5, and 1:1 returned to a gel-type after cooling. This was attributed to the decrease in G/P ratio, which reduced the supramolecular interactions, increasing hydrophilic and hydrogen bonding interactions from the gelatin backbones when rising the temperature.

Next, the Gel-Phe polymers with the ratios of 1:15, 1:10, 1:5, and 1:1 were mixed with GelMA through the same weight percentage in water. Noticeably, compared with pure Gel-Phe, the Gel-Phe with the ratios of 1:15, 1:10, 1:5, and 1:1 mixed with GelMA showed good dispersion and had no gel formation after a cooling process (Figure 2c). This reasonably suggests that the chain of GelMA contributed a high affinity to the gelatin residues on the Gel-Phe backbone at the low G/P molar ratios. Moreover, these GelMA/Gel-Phe solutions could also be crosslinked and form hydrogels after UV light exposure (Figure 2d,e).

To evaluate the formation of hydrogels, the Gel-Phe polymers with the G/P ratios of 1:1, 1:5, 1:10, and 1:15 were mixed with GelMA and 0.5% PI. Both final concentrations of Gel-Phe and GelMA in the solutions were equal. Then, the hybrid GelMA/Gel-Phe solutions were tested for gel formation behavior by exposure to 15, 30, and 60 s of UV light (800 mW/cm^2^) [31]. As shown in Figure 2f, the hydrogels were able to form after UV exposure, and no significant difference in morphology could be observed in all GelMA/Gel-Phe conditions. These results indicate that the Gel-Phe polymers with the G/P ratios of 1:1, 1:5, 1:10, and 1:15 were well mixed with GelMA, showing that the mixed GelMA/GePhe solution was able to be used as a bioadhesive agent.

### 3.3. Evaluation of the Injectability and Mechanical Properties of Hybrid GelMA/Gel-Phe Bioadhesive

**Figure 3:**
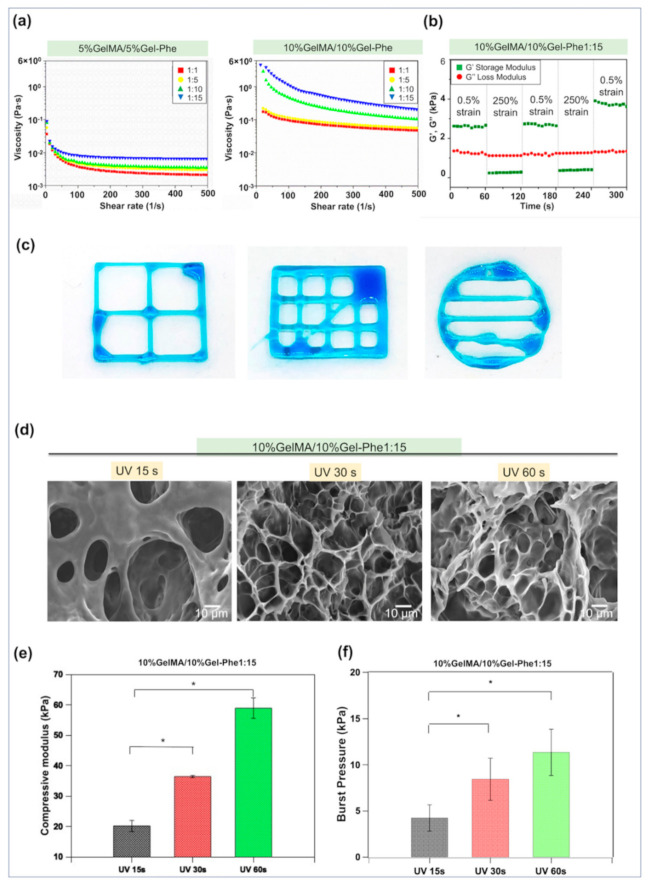
Evaluation of the injectable and mechanical properties of hybrid GelMA/Gel-Phe hydrogels. (**a**) The shear-thinning behavior and (**b**) the oscillatory strain sweep test between a low (0.5%) and high (250%) strain of the GelMA/Gel-Phe solutions. (**c**) The injectability testing of 10%GelMA/10%Gel-Phe1:15 solution through an extrusion bioprinter. (**d**) The SEM morphology, (**e**) compressive modulus, and (**f**) the in vitro burst pressure of hybrid 10%GelMA/10%Gel-Phe1:15 hydrogels after exposure to different UV times. *: *p* < 0.05.

Next, we tested the rheological properties of GelMA/Gel-Phe solutions to evaluate the injectability of the hybrid solution through a linearly ramped shear rate and an oscillatory strain sweep model. Despite Irgacure 2959 being the most biocompatible UV photoinitiator for various cell types, some cells were still sensitive to the Irgacure 2959 [48]. Therefore, as the results from Figure 2f show, 0.5% PI was selected as the condition for the following experiments according to our previous study [31]. The apparent viscosity of the GelMA/Gel-Phe solutions in all groups decreased following the increase in shear rate from 0 s^−1^ to 500 s^−1^, indicating that the GelMA/Gel-Phe solutions exhibited a non-Newtonian fluidic behavior (Figure 3a). Compared to the 5%GelMA/5%Gel-Phe groups, the presence of 10% GelMA/10% Gel-Phe enhanced the internal molecular bonding interactions and then raised the initial viscosity of the solutions that offered the GelMA/Gel-Phe solutions a slow-flowing behavior for injection. In this analysis, we also observed that the Gel-Phe polymer with a high G/P ratio contributed to increasing the viscosity of the GelMA/Gel-Phe solutions, especially the G/P ratio of 1:15, which was attributed to the supramolecular interactions among the phenyl isothiocyanate group, gelatin, and GeMA chains. Therefore, we suggested that the group 10%GelMA/10%Gel-Phe1:15/0.5%PI may be a candidate for injection, and this group was selected for the following analysis.

Next, the 10%GelMA/10%Gel-Phe1:15/0.5%PI solution was further evaluated its solid-like to a liquid-like state transition behavior (Figure 3b) using an oscillatory strain sweep model. Under the 0.5% strain period, the GelMA/Gel-Phe solution showed the storage modulus (G’) over the loss modulus (G”), indicating a stable solid phase within the first 60 s. In contrast, under the 250% strain period, a rapid phase transition from solid to liquid was observed because of the G” over the G’. Subsequently, the GelMA/Gel-Phe solution presented a solid state from liquid when the strain returned to 0.5%, suggesting that the high physical internal molecular interactions in the GelMA/Gel-Phe solution caused a good mechanical recovery. Next, we performed a printing test on the GelMA/Gel-Phe solution to assess their injectability through an extrusion bioprinter. As shown in Figure 3c, the solution containing the 10% GelMA/10% Gel-Phe1:15/0.5% PI solution enabled us to print window, grid, and circle shapes, showing a fluid-like gel behavior with injectability, which was consistent with that in rheological testing. As the results from Figure 2f and Figure 3a,b show, the 10%GelMA/10%Gel-Phe1:15 solution possessed injectability and could form a crosslinked hydrogel through UV light exposure.

Furthermore, the microstructure of crosslinked GelMA/Gel-Phe hydrogels formed by different UV exposure times is characterized in Figure 3d. Compared with the pure GelMA hydrogel (Figure S3), the porous density in the GelMA/Gel-Phe hydrogels gradually increased when UV exposure times increased. The phenomenon is attributed to the slight phase separation between the intrinsic immiscibility of the chemical crosslink of GelMA chains and the hydrophobic phenyl groups on Gel-Phe and GelMA polymers [49].

Additionally, the hydrophilic segment of Gel-Phe chains also may also form an interpenetrating polymer network with the GelMA chains, which can enhance the mechanical properties of the crosslinked GelMA/Gel-Phe hydrogels (Figure 3e). Moreover, one of the crucial parameters for hemostatic agents is the adhesive ability, which can avoid the movement of the adhesive on the bleeding site. Next, we investigated the adhesive ability of crosslinked GelMA/Gel-Phe hydrogels via the burst-pressure test (Figure 3f). The burst pressure of crosslinked 10%GelMA/10%Gel-Phe1:15 hydrogel achieved 11.3 kPa ± 2.5 kPa after the 60 s of UV exposure, which was closed to normal blood pressure and may withstand the blood pressure during the hemostatic procedure [32]. Therefore, these results indicated that the 10%GelMA/10%Gel-Phe1:15 hydrogel treated with UV light exposure might possess potential use as a bioadhesive.

### 3.4. In Vitro Hemostastic Models for Examination of the GelMA/Gel-Phe Bioadhesives

**Figure 4:**
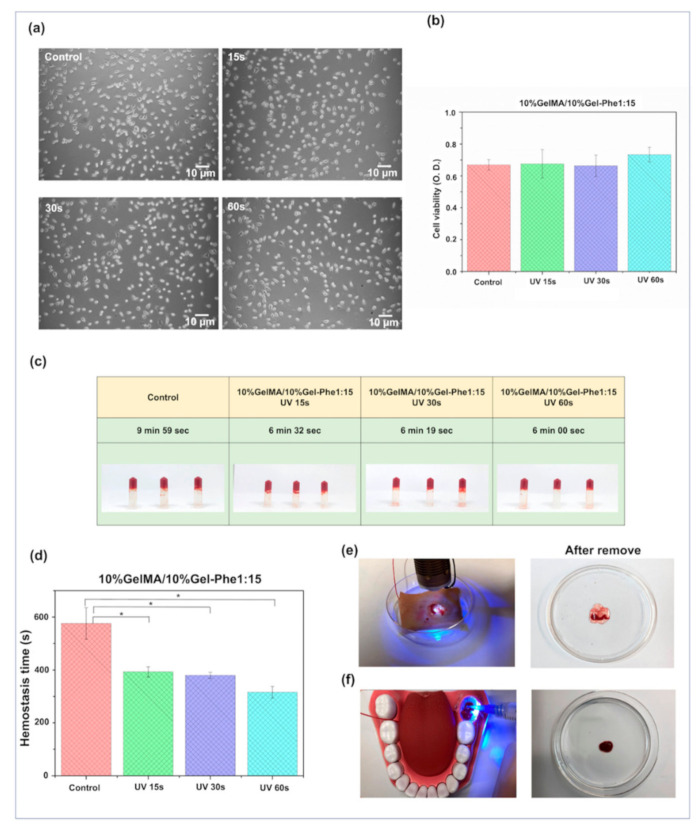
Validation of the hemostatic function of GelMA/Gel-Phe bioadhesives. (**a**) The cell morphology and (**b**) cell viability of the L929 cells treated with the extraction medium from the GelMA/Gel-Phe bioadhesives with different UV exposure times. (**c**) The in vitro blood clotting test and (**d**) the hemostasis times of GelMA/Gel-Phe bioadhesives with different UV exposure times. * *p* < 0.05. (**e**) The in vitro porcine skin and (**f**) tooth extraction bleeding models for mimicking the 10%GelMA/10%Gel-Phe1:15 bioadhesive under a blood perfusion. The images show that the bioadhesive had a rapid hemostatic effect within the 60 s of UV light exposure and could be easily removed from the bleeding site after crosslinking.

The cytotoxic effect of bioadhesives on cells is another indispensable parameter that should be investigated because bioadhesives usually directly contact living tissues during the hemostatic process. As shown in Figure 4a,b, the 10%GelMA/10%Gel-Phe1:15 bioadhesives formed by different UV exposure times were used to culture a fibroblast cell line, L929, to evaluate the cytotoxicity of GelMA/Gel-Phe bioadhesives according to the ISO 10993-5 standard protocol [35]. The hybrid GelMA/Gel-Phe bioadhesives were immersed in the culture medium for 1 day, and the extraction medium was subsequently used to culture fibroblasts, which were pre-cultured overnight for adhesion on the culture plate. Compared with the control group (i.e., cells treated with fresh medium), the cell morphology and cell viability showed no significant differences among the GelMA/Gel-Phe bioadhesives formed by different UV exposure times, indicating that the GelMA/Gel-Phe bioadhesives had no cytotoxicity for the cultured cells.

Next, the in vitro clot formation model was used to validate the blood coagulation ability of the crosslinked GelMA/Gel-Phe bioadhesives (Figure 4c,d). We first added 0.02 M calcium ions to the commercial fresh sheep blood (i.e., control group), and the average initial blood clotting time was 575 s ± 69 s. Compared to the control group, the average blood clotting time of GelMA/Gel-Phe bioadhesive treated with the 15 s UV exposure time promoted rapid coagulation and decreased clotting time by 32% (392 s ± 19 s). These results suggested that the increase in porous density in the GelMA/Gel-Phe bioadhesive contributed to a high surface area, promoting more bioactive segments in whole blood components absorbed in the bioadhesive to reduce the clotting time. Furthermore, when the bioadhesive was exposed to 60 s of UV light, the clotting time was reduced to 54% of the control group time (315 s ± 22 s). Here, the synergistic effects of increased porous structure and urea-like and amine groups in the GelMA/Gel-Phe bioadhesive may play a catalytic role in adsorbing the coagulation factors and the active blood clotting cascade, and then promote fibrin polymerization to enhance the hemostatic efficiency [50,51,52,53,54]. Compared with blood without any treatment, many agents and biomaterials could enhance the blood clotting time [55,56]. For instance, the thrombin agents and clay-based gels contributed to the most rapid effect, reducing the 80% clotting time [56,57]. Additionally, foam-type materials such as Instat or Gelfoam could reduce the clotting time by 20% [56,58]. Additionally, pure hydrogel-type materials, such as chitosan or gelatin hydrogels, without the addition of enzyme or clay enabled a decrease in coagulation time of around 20% [55,59]. In this study, the 10%GelMA/10%Gel-Phe1:15/0.5%PI bioadhesive with 60 s of UV light exposure could reduce the blood clotting time by 45%. Compared with the other materials, although the hemostatic efficiency of GelMA/Gel-Phe bioadhesive did not reach that of nanoclay-based materials or thrombin-based agents, the GelMA/Gel-Phe bioadhesive contributed a sealant-like property and a medium clotting time. These performances indicated that the GelMA/Gel-Phe bioadhesive possesses potential applications as an injectable hemostatic material.

Lastly, to validate the blood clotting effect of the GelMA/Gel-Phe bioadhesive on the dynamic bleeding models. As shown in Figure 4e,f, the in vitro skin bleeding model and dental bleeding models were used as the preliminary testing models to mimic skin bleeding and bleeding after tooth extraction. According to the injectability from Figure 3a–c, the un-crosslinked 10%GelMA/10%Gel-Phe1:15 bioadhesive extruded through the nozzle, indicating that the bioadhesive was suitable for commercial syringes and has a potential to be used as a portable hemostatic agent. Therefore, the un-crosslinked GelMA/Gel-Phe bioadhesive was directly extruded on the bleeding site where a continuous bloodstream was perfused (flow rate: 400 μL/min^−1^). Afterward, the bioadhesive was crosslinked on the bleeding site by treatment with 60 s of UV exposure. (Figure 4e,f, Videos S1 and S2). In the skin and tooth extraction bleeding models (Figure 4e,f), no blood leaking out from the edge of 10%GelMA/10%Gel-Phe1:15 bioadhesive was observed during the photo-crosslinking period.

Additionally, the crosslinked bioadhesive could be removed from the porcine skin sheet and the tooth hole (Figure 4e,f), showing that the bioadhesive crosslinked on these wound sites may avoid a secondary injury after removing the bioadhesive. These results suggested that the GelMA/Gel-Phe enabled the rapid absorption of blood and then accelerated the hemostasis effect during the blood diffusion in the bioadhesive. Moreover, these results also confirmed that the injectable GelMA/Gel-Phe bioadhesive presented a good burst pressure (Figure 3f), allowing for the adhesion of the bioadhesive on the bleeding site and offering hemostatic ability after crosslinking via the UV light exposure. Overall, these data show the potential feasibility of the injectable GelMA/Gel-Phe bioadhesive for hemostatic applications in tooth extraction or oral/dental surgical process.

Furthermore, although our results show the GelMA/Gel-Phe bioadhesive with potential for oral/dental hemostatic applications, the GelMA/Gel-Phe bioadhesive with only physical interactions may limit the blood clotting time. Additionally, the GelMA/Gel-Phe bioadhesive may have no functions in enhancing the wound healing of oral tissues after surgery. Our study also provides future perspectives to overcome the current limitations and extend the applications of our concept. First, the addition of chemical compounds in the GelMA/Gel-Phe bioadhesives may enhance their hemostatic efficiency. For example, clay rectorite was shown to have a highly hemostatic effect in our previous study [35]. As a result, the combination of clay rectorite and the GelMA/Gel-Phe may synergistically decrease blood clotting time. In addition, other compounds, such thrombin, fibrin, or functional materials, are also candidates as additives for enhancing blood clotting efficiency. Moreover, another concept is that the GelMA/Gel-Phe bioadhesive could be used as a wound healing scaffold for oral tissue engineering. As our results show, GelMA/Gel-Phe had injectability–printability, shape–plasticity, and regulable mechanical properties. Additionally, GelMA-based materials have been confirmed for their biocompatibility and biodegradability in mucosa tissue [60]. Therefore, we envision that the GelMA/Gel-Phe bioadhesive could mix with growth factors, anti-inflammatory drugs, or an extracellular matrix to develop versatile scaffolds for wound healing or tissue reconstruction in oral tissue engineering.

## 4. Conclusions

In this study, we developed a proof-of-concept photo-crosslinkable bioadhesive based on the Gel-Phe polymer and the bioactive GelMA polymer for oral/dental hemostatic applications. The hybrid properties and crosslinkable mechanisms from both Gel-Phe and GelMA polymers offered accessible modulable injectability, mechanical properties, and adhesivity. The GelMA/Gel-Phe bioadhesive can be rapidly cured in situ through photo-crosslinking and rapidly absorb blood, as validated through the dynamic bleeding tests. Therefore, this novel class of GelMA/Gel-Phe bioadhesive with injectability and immediate hemostatic effect has the potential to be a fast crosslinkable hemostatic agent for irregular wounds in oral/dental surgical procedures.

## Supplementary Materials

The following are available online at https://www.mdpi.com/article/10.3390/polym13142386/s1, Figure S1: FTIR spectrum and ^1^H-NMR spectrum of GelMA and gelatin polymers; Figure S2: The morphology of Gel-Phe hydrogels synthesized with different G/P ratios; Figure S3: The SEM morphology of GelMA hydrogels without the addition of Gel-Phe; Video S1: Hemostatic test using the in vitro porcine skin bleeding model; Video S2: Hemostatic test using the in vitro dental bleeding model.
