# Supplementary material for: An Injectable Hybrid Gelatin Methacryloyl (GelMA)/Phenyl Isothiocyanate-Modified Gelatin (Gel-Phe) Bioadhesive for Oral/Dental Hemostasis Applications
Source: Polymers (Basel). 2021 Jul 20;13(14):2386. doi: 10.3390/polym13142386 (PMC8309571; doi:10.3390/polym13142386)
Supplement: Supplementary file 1 [file polymers-13-02386-s001.zip › Supporting Information-2021-0505.pdf]

Supporting Information

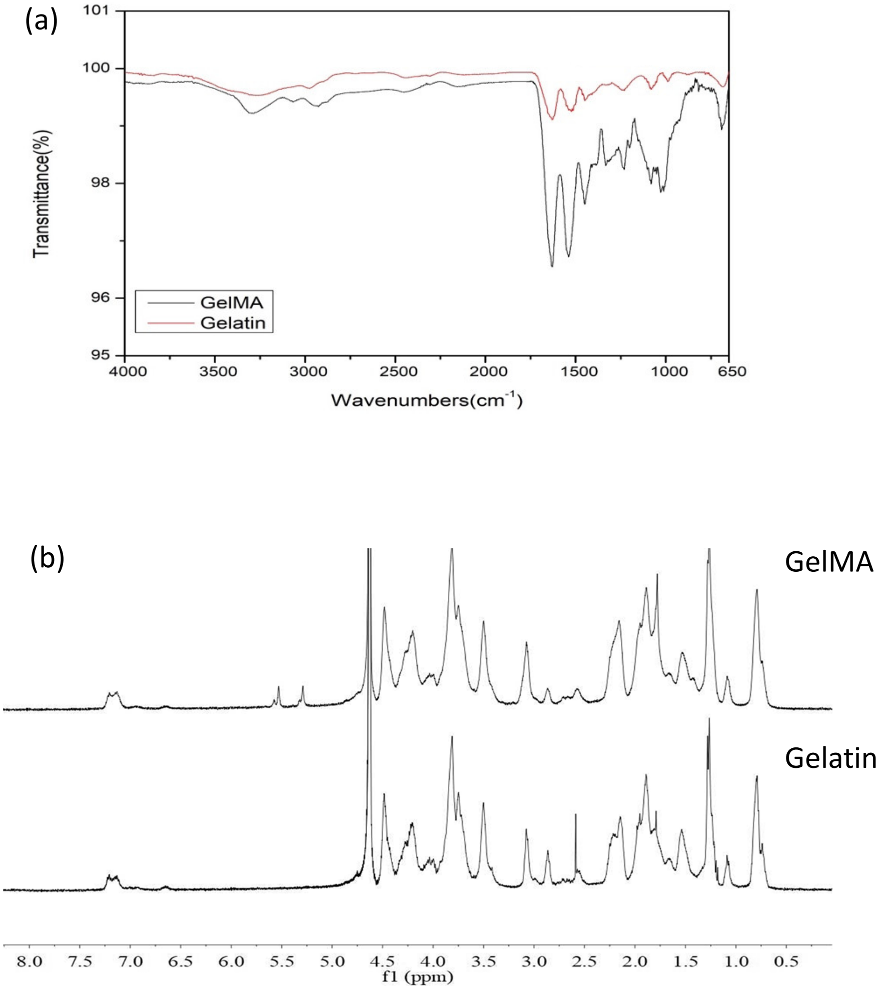


Supporting Figure S1. (a) FTIR spectrum of GelMA and gelatin polymers showing characteristic relative peaks of functional groups. (b) $^{1}$H-NMR spectrum of GelMA and gelatin polymers, confirming the chemical modification of gelatin backbones with methacrylic anhydride.

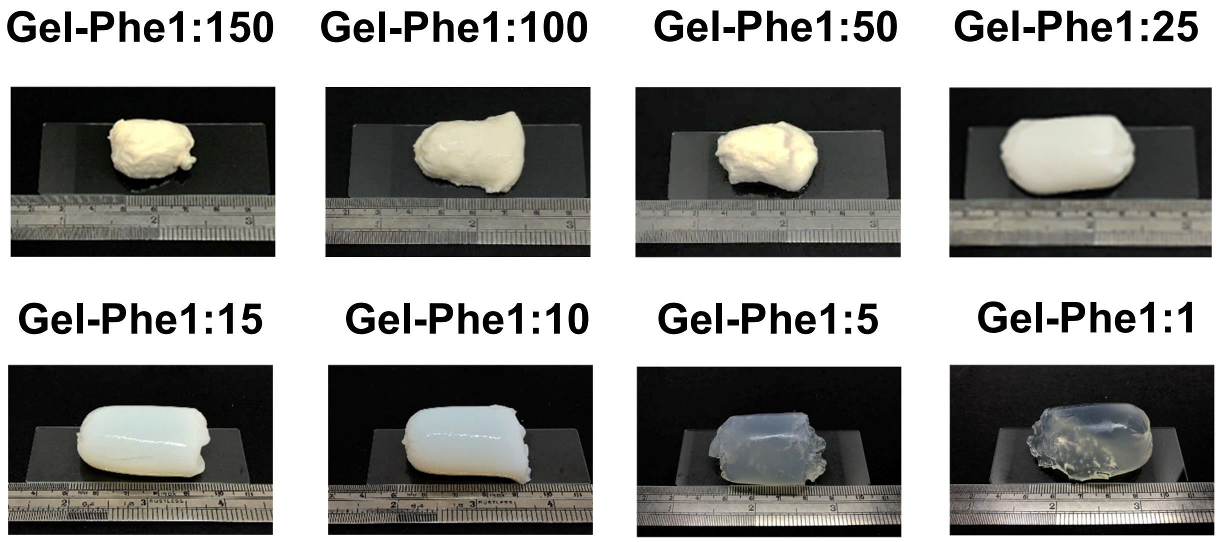


Supporting Figure S2. The morphology of Gel-Phe hydrogels synthesized with different G/P ratios after dialysis process at room temperature.

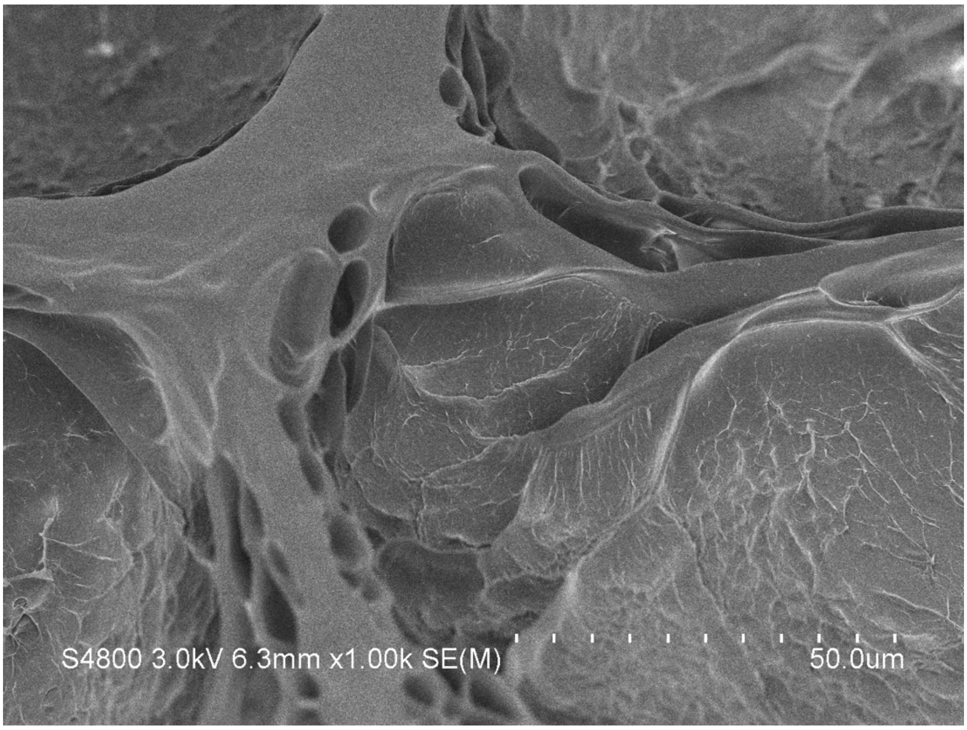


Supporting Figure S3. The SEM morphology of GelMA hydrogels without the addition of Gel-Phe after exposing with 60s of UV light..
